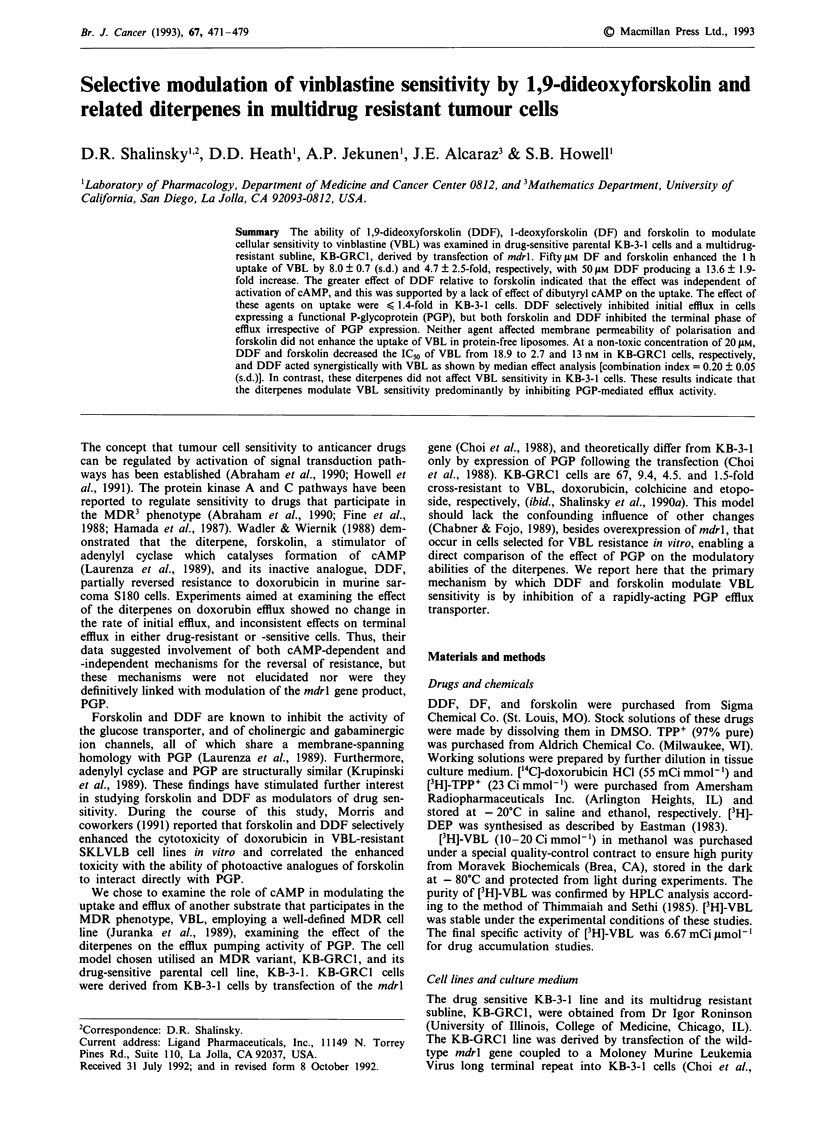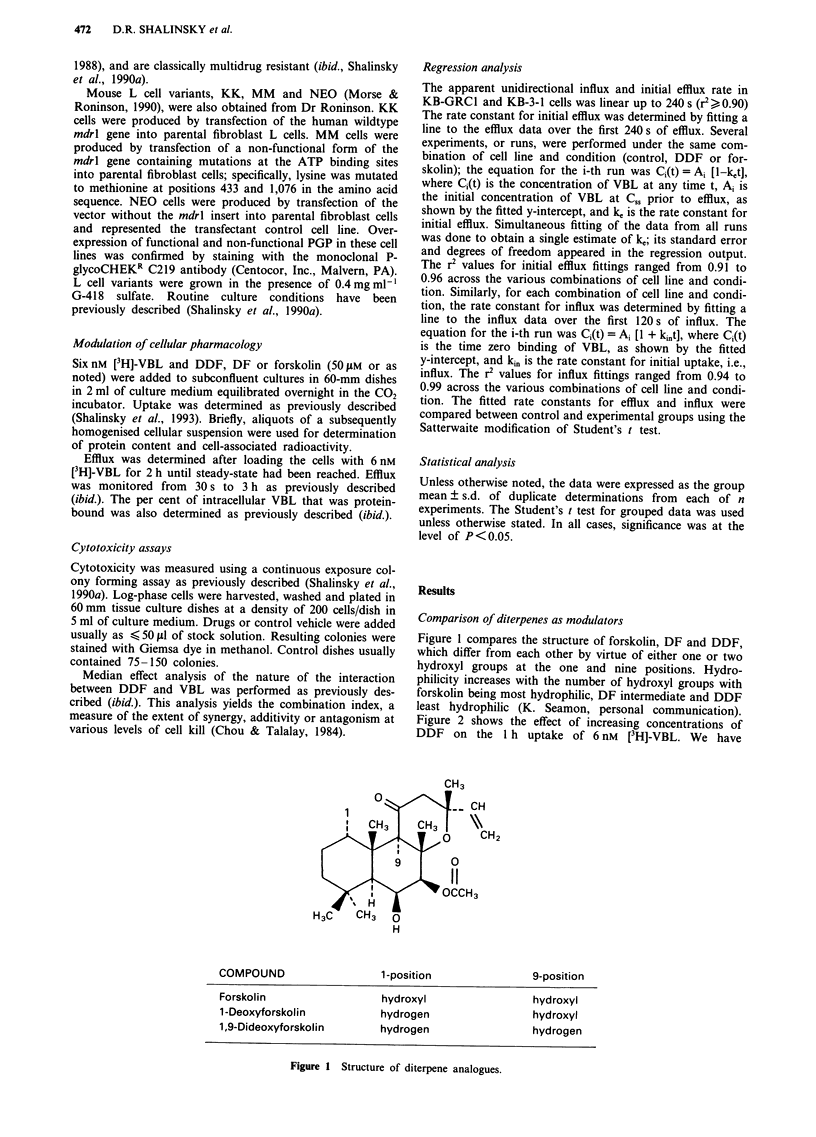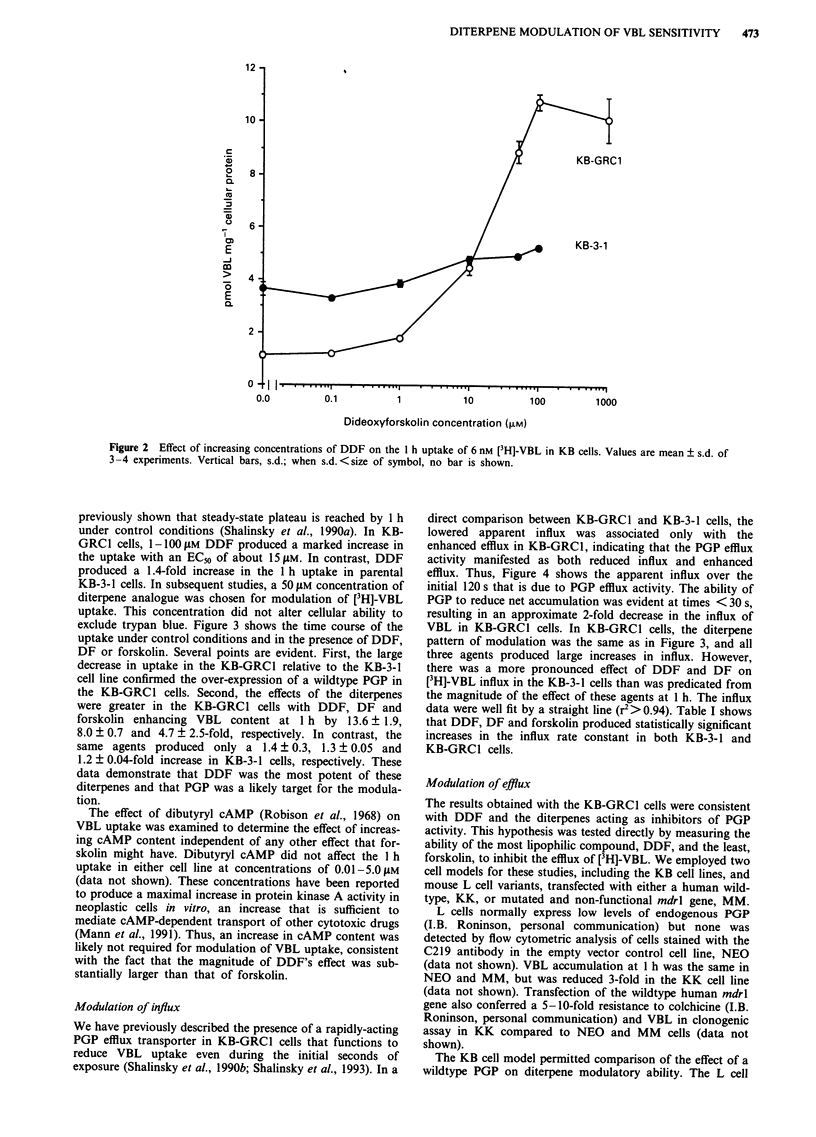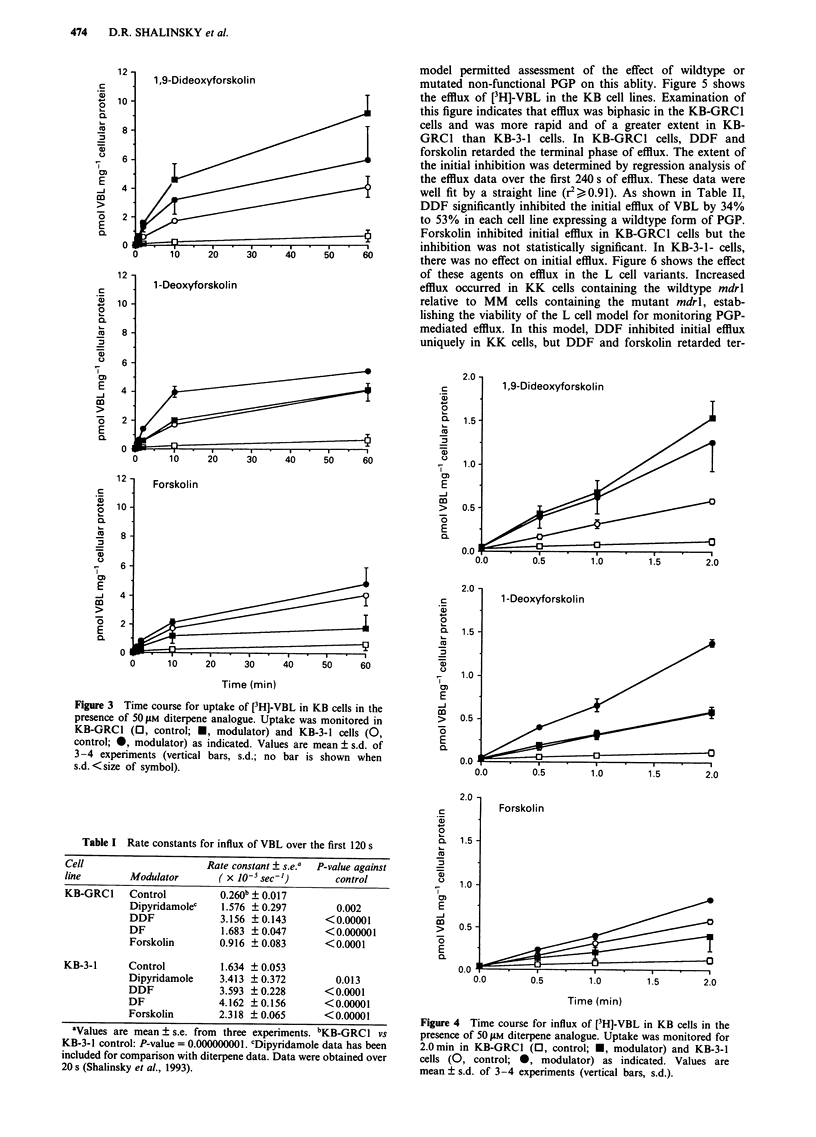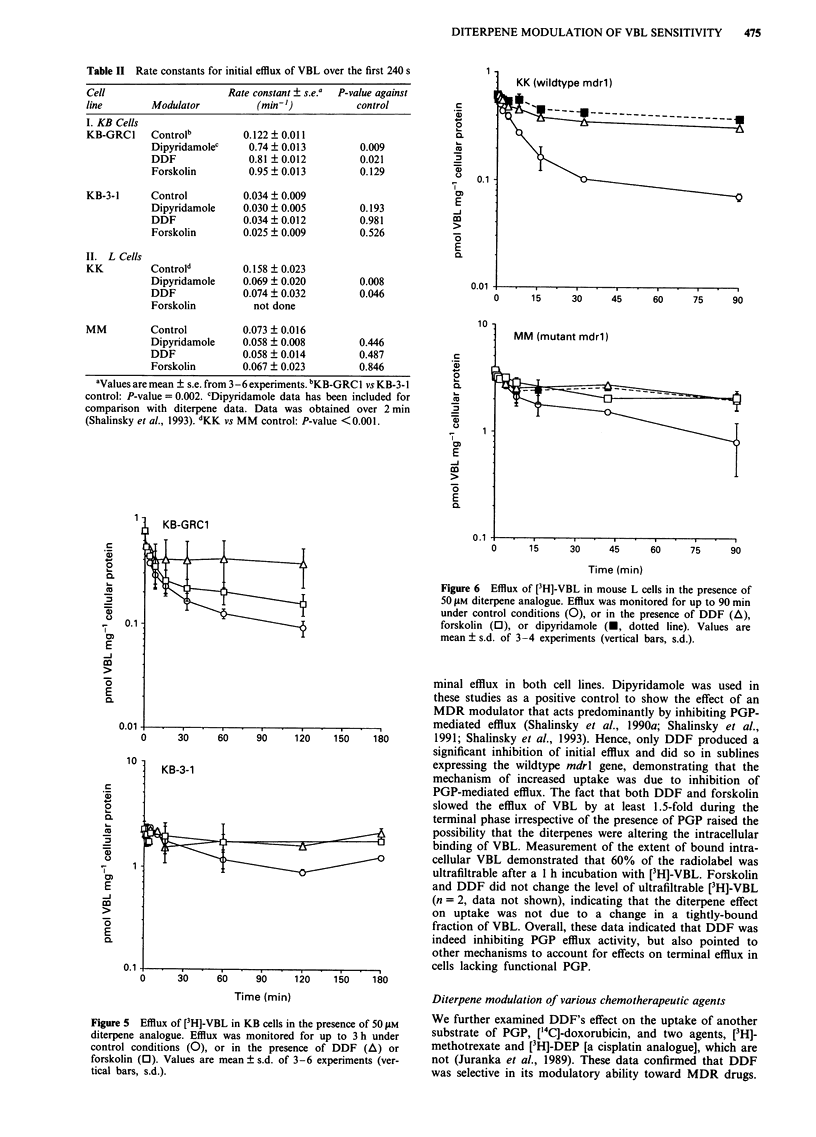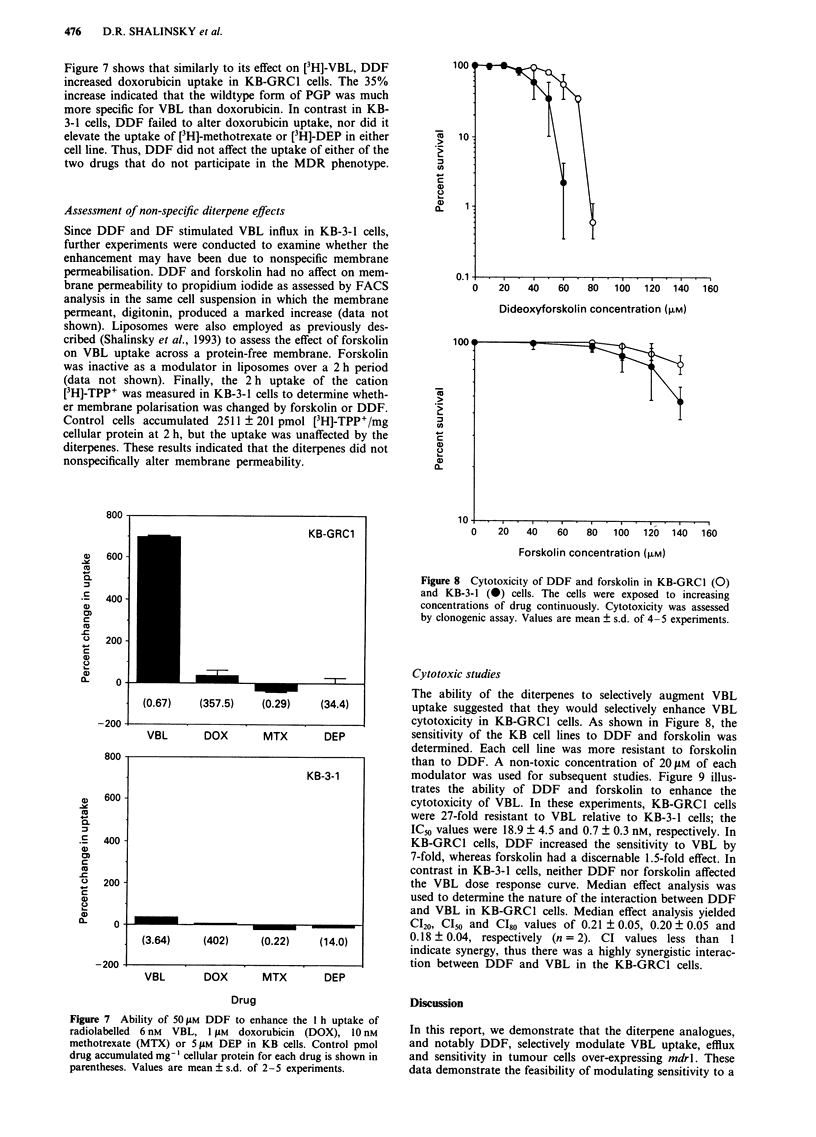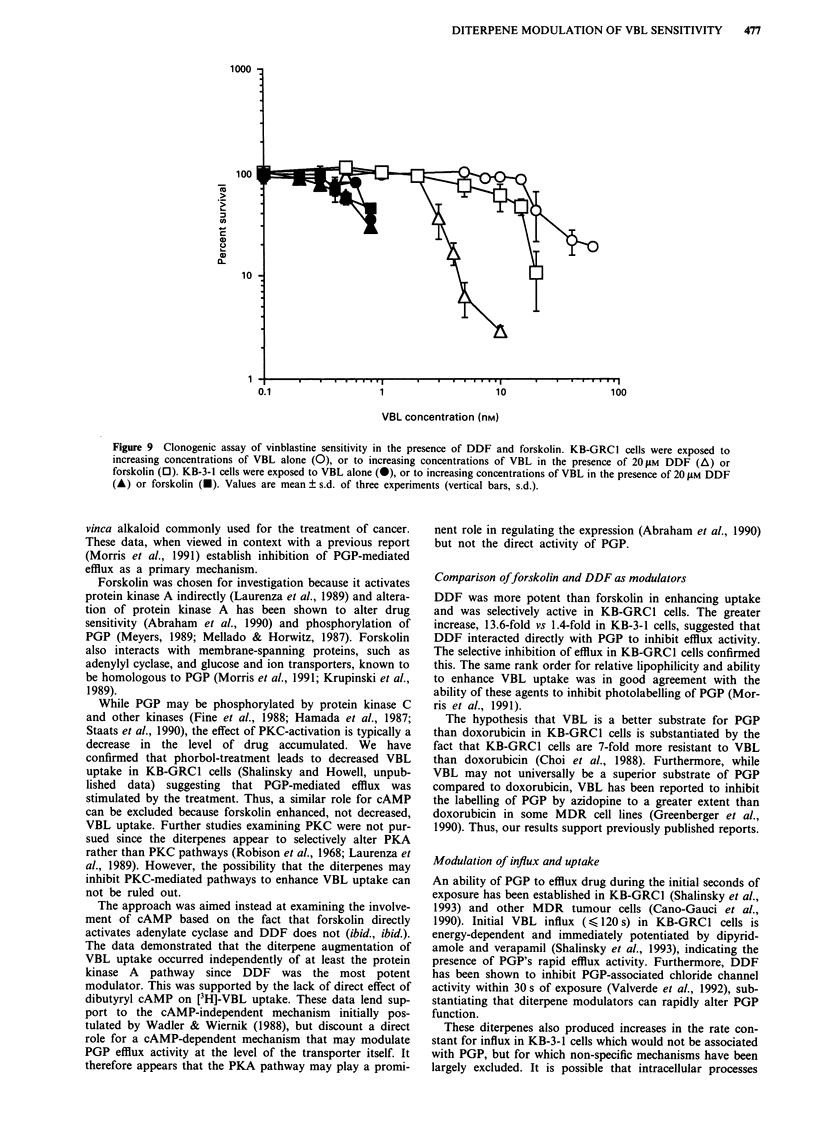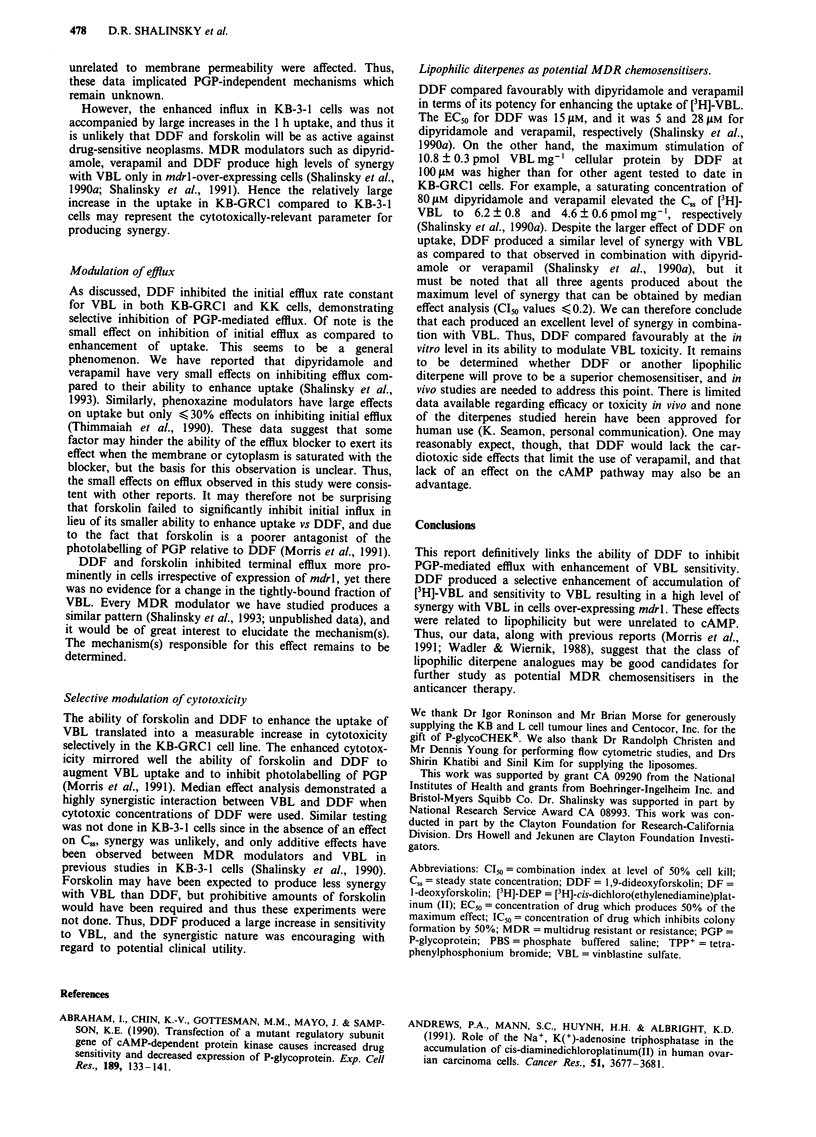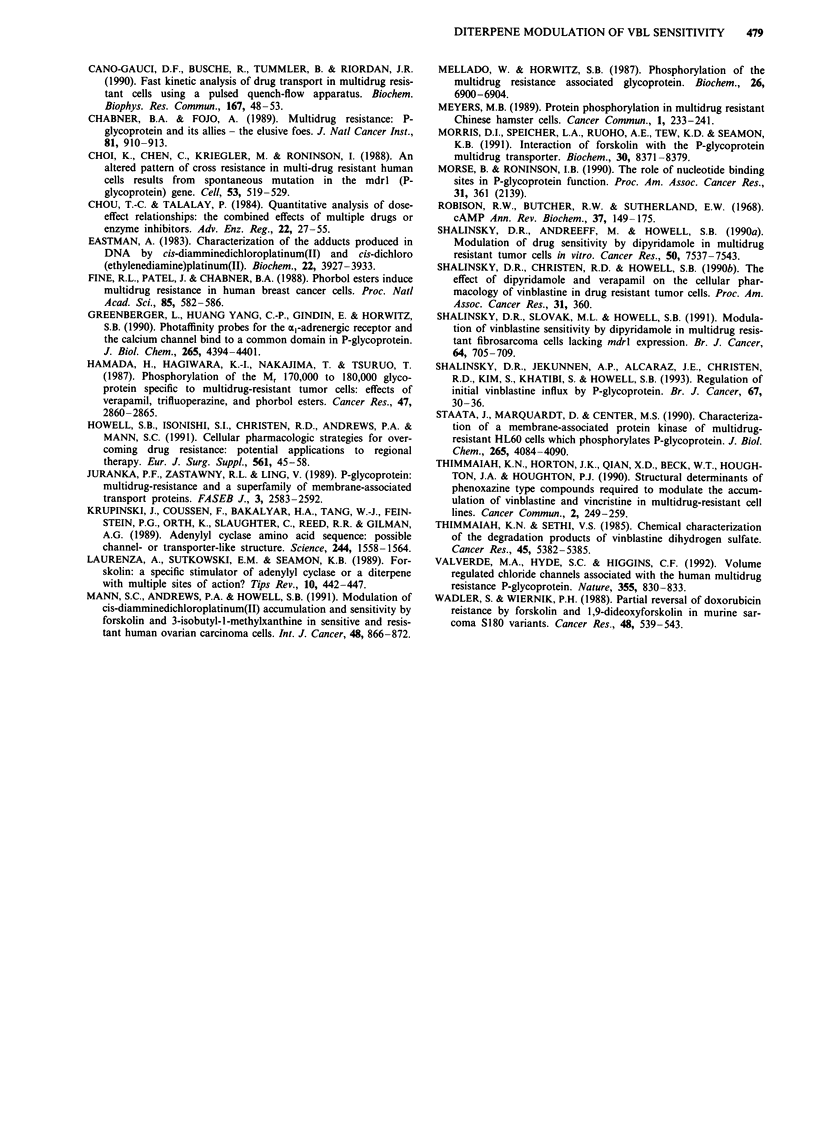# Selective modulation of vinblastine sensitivity by 1,9-dideoxyforskolin and related diterpenes in multidrug resistant tumour cells.

**DOI:** 10.1038/bjc.1993.89

**Published:** 1993-03

**Authors:** D. R. Shalinsky, D. D. Heath, A. P. Jekunen, J. E. Alcaraz, S. B. Howell

**Affiliations:** Department of Medicine and Cancer Center, University of California, San Diego, La Jolla 92093-0812.

## Abstract

The ability of 1,9-dideoxyforskolin (DDF), 1-deoxyforskolin (DF) and forskolin to modulate cellular sensitivity to vinblastine (VBL) was examined in drug-sensitive parental KB-3-1 cells and a multidrug-resistant subline, KB-GRC1, derived by transfection of mdr1. Fifty microM DF and forskolin enhanced the 1 h uptake of VBL by 8.0 +/- 0.7 (s.d.) and 4.7 +/- 2.5-fold, respectively, with 50 microM DDF producing a 13.6 +/- 1.9-fold increase. The greater effect of DDF relative to forskolin indicated that the effect was independent of activation of cAMP, and this was supported by a lack of effect of dibutyryl cAMP on the uptake. The effect of these agents on uptake were < or = 1.4-fold in KB-3-1 cells. DDF selectively inhibited initial efflux in cells expressing a functional P-glycoprotein (PGP), but both forskolin and DDF inhibited the terminal phase of efflux irrespective of PGP expression. Neither agent affected membrane permeability of polarisation and forskolin did not enhance the uptake of VBL in protein-free liposomes. At a non-toxic concentration of 20 microM, DDF and forskolin decreased the IC50 of VBL from 18.9 to 2.7 and 13 nM in KB-GRC1 cells, respectively, and DDF acted synergistically with VBL as shown by median effect analysis [combination index = 0.20 +/- 0.05 (s.d.)]. In contrast, these diterpenes did not affect VBL sensitivity in KB-3-1 cells. These results indicate that the diterpenes modulate VBL sensitivity predominantly by inhibiting PGP-mediated efflux activity.


Br. J. Cancer (1993), 67, 471–479 © Macmillan Press Ltd., 1993

### Selective modulation of vinblastine sensitivity by 1,9-dideoxyforskolin and related diterpenes in multidrug resistant tumour cells

D.R. Shalinsky[1,2], D.D. Heath[1], A.P. Jekunen[1], J.E. Alcaraz[3] & S.B. Howell[1]

[1]*Laboratory of Pharmacology, Department of Medicine and Cancer Center 0812, and* [3]*Mathematics Department, University of California, San Diego, La Jolla, CA 92093-0812, USA.*

**Summary** The ability of 1,9-dideoxyforskolin (DDF), 1-deoxyforskolin (DF) and forskolin to modulate cellular sensitivity to vinblastine (VBL) was examined in drug-sensitive parental KB-3-1 cells and a multidrug-resistant subline, KB-GRC1, derived by transfection of *mdr*1. Fifty μM DF and forskolin enhanced the 1 h uptake of VBL by 8.0 ± 0.7 (s.d.) and 4.7 ± 2.5-fold, respectively, with 50 μM DDF producing a 13.6 ± 1.9-fold increase. The greater effect of DDF relative to forskolin indicated that the effect was independent of activation of cAMP, and this was supported by a lack of effect of dibutyryl cAMP on the uptake. The effect of these agents on uptake were ⩽ 1.4-fold in KB-3-1 cells. DDF selectively inhibited initial efflux in cells expressing a functional P-glycoprotein (PGP), but both forskolin and DDF inhibited the terminal phase of efflux irrespective of PGP expression. Neither agent affected membrane permeability of polarisation and forskolin did not enhance the uptake of VBL in protein-free liposomes. At a non-toxic concentration of 20 μM, DDF and forskolin decreased the $IC_{50}$ of VBL from 18.9 to 2.7 and 13 nM in KB-GRC1 cells, respectively, and DDF acted synergistically with VBL as shown by median effect analysis [combination index = 0.20 ± 0.05 (s.d.)]. In contrast, these diterpenes did not affect VBL sensitivity in KB-3-1 cells. These results indicate that the diterpenes modulate VBL sensitivity predominantly by inhibiting PGP-mediated efflux activity.

The concept that tumour cell sensitivity to anticancer drugs can be regulated by activation of signal transduction pathways has been established (Abraham *et al.*, 1990; Howell *et al.*, 1991). The protein kinase A and C pathways have been reported to regulate sensitivity to drugs that participate in the MDR[3] phenotype (Abraham *et al.*, 1990; Fine *et al.*, 1988; Hamada *et al.*, 1987). Wadler & Wiernik (1988) demonstrated that the diterpene, forskolin, a stimulator of adenylyl cyclase which catalyses formation of cAMP (Laurenza *et al.*, 1989), and its inactive analogue, DDF, partially reversed resistance to doxorubicin in murine sarcoma S180 cells. Experiments aimed at examining the effect of the diterpenes on doxorubin efflux showed no change in the rate of initial efflux, and inconsistent effects on terminal efflux in either drug-resistant or -sensitive cells. Thus, their data suggested involvement of both cAMP-dependent and -independent mechanisms for the reversal of resistance, but these mechanisms were not elucidated nor were they definitively linked with modulation of the *mdr*1 gene product, PGP.

Forskolin and DDF are known to inhibit the activity of the glucose transporter, and of cholinergic and gabaminergic ion channels, all of which share a membrane-spanning homology with PGP (Laurenza *et al.*, 1989). Furthermore, adenylyl cyclase and PGP are structurally similar (Krupinski *et al.*, 1989). These findings have stimulated further interest in studying forskolin and DDF as modulators of drug sensitivity. During the course of this study, Morris and coworkers (1991) reported that forskolin and DDF selectively enhanced the cytotoxicity of doxorubicin in VBL-resistant SKLVLB cell lines *in vitro* and correlated the enhanced toxicity with the ability of photoactive analogues of forskolin to interact directly with PGP.

We chose to examine the role of cAMP in modulating the uptake and efflux of another substrate that participates in the MDR phenotype, VBL, employing a well-defined MDR cell line (Juranka *et al.*, 1989), examining the effect of the diterpenes on the efflux pumping activity of PGP. The cell model chosen utilised an MDR variant, KB-GRC1, and its drug-sensitive parental cell line, KB-3-1. KB-GRC1 cells were derived from KB-3-1 cells by transfection of the *mdr*1 gene (Choi *et al.*, 1988), and theoretically differ from KB-3-1 only by expression of PGP following the transfection (Choi *et al.*, 1988). KB-GRC1 cells are 67, 9.4, 4.5. and 1.5-fold cross-resistant to VBL, doxorubicin, colchicine and etoposide, respectively, (*ibid.*, Shalinsky *et al.*, 1990*a*). This model should lack the confounding influence of other changes (Chabner & Fojo, 1989), besides overexpression of *mdr*1, that occur in cells selected for VBL resistance *in vitro*, enabling a direct comparison of the effect of PGP on the modulatory abilities of the diterpenes. We report here that the primary mechanism by which DDF and forskolin modulate VBL sensitivity is by inhibition of a rapidly-acting PGP efflux transporter.

[2]Correspondence: D.R. Shalinsky.
Current address: Ligand Pharmaceuticals, Inc., 11149 N. Torrey Pines Rd., Suite 110, La Jolla, CA 92037, USA.
Received 31 July 1992; and in revised form 8 October 1992.

### Materials and methods

### *Drugs and chemicals*

DDF, DF, and forskolin were purchased from Sigma Chemical Co. (St. Louis, MO). Stock solutions of these drugs were made by dissolving them in DMSO. $TPP^+$ (97% pure) was purchased from Aldrich Chemical Co. (Milwaukee, WI). Working solutions were prepared by further dilution in tissue culture medium. [$^{14}C$]-doxorubicin HCl (55 mCi $mmol^{-1}$) and [$^3H$]-$TPP^+$ (23 Ci $mmol^{-1}$) were purchased from Amersham Radiopharmaceuticals Inc. (Arlington Heights, IL) and stored at − 20°C in saline and ethanol, respectively. [$^3H$]-DEP was synthesised as described by Eastman (1983).

[$^3H$]-VBL (10–20 Ci $mmol^{-1}$) in methanol was purchased under a special quality-control contract to ensure high purity from Moravek Biochemicals (Brea, CA), stored in the dark at − 80°C and protected from light during experiments. The purity of [$^3H$]-VBL was confirmed by HPLC analysis according to the method of Thimmaiah and Sethi (1985). [$^3H$]-VBL was stable under the experimental conditions of these studies. The final specific activity of [$^3H$]-VBL was 6.67 mCi $\mu mol^{-1}$ for drug accumulation studies.

### *Cell lines and culture medium*

The drug sensitive KB-3-1 line and its multidrug resistant subline, KB-GRC1, were obtained from Dr Igor Roninson (University of Illinois, College of Medicine, Chicago, IL). The KB-GRC1 line was derived by transfection of the wild-type *mdr*1 gene coupled to a Moloney Murine Leukemia Virus long terminal repeat into KB-3-1 cells (Choi *et al.*,

1988), and are classically multidrug resistant (*ibid.*, Shalinsky *et al.*, 1990*a*).

Mouse L cell variants, KK, MM and NEO (Morse & Roninson, 1990), were also obtained from Dr Roninson. KK cells were produced by transfection of the human wildtype *mdr*1 gene into parental fibroblast L cells. MM cells were produced by transfection of a non-functional form of the *mdr*1 gene containing mutations at the ATP binding sites into parental fibroblast cells; specifically, lysine was mutated to methionine at positions 433 and 1,076 in the amino acid sequence. NEO cells were produced by transfection of the vector without the *mdr*1 insert into parental fibroblast cells and represented the transfectant control cell line. Overexpression of functional and non-functional PGP in these cell lines was confirmed by staining with the monoclonal P-glycoCHEK$^R$ C219 antibody (Centocor, Inc., Malvern, PA). L cell variants were grown in the presence of 0.4 mg ml$^{-1}$ G-418 sulfate. Routine culture conditions have been previously described (Shalinsky *et al.*, 1990*a*).

### *Modulation of cellular pharmacology*

Six nM [$^3$H]-VBL and DDF, DF or forskolin (50 μM or as noted) were added to subconfluent cultures in 60-mm dishes in 2 ml of culture medium equilibrated overnight in the $CO_2$ incubator. Uptake was determined as previously described (Shalinsky *et al.*, 1993). Briefly, aliquots of a subsequently homogenised cellular suspension were used for determination of protein content and cell-associated radioactivity.

Efflux was determined after loading the cells with 6 nM [$^3$H]-VBL for 2 h until steady-state had been reached. Efflux was monitored from 30 s to 3 h as previously described (*ibid.*). The per cent of intracellular VBL that was protein-bound was also determined as previously described (*ibid.*).

### *Cytotoxicity assays*

Cytotoxicity was measured using a continuous exposure colony forming assay as previously described (Shalinsky *et al.*, 1990*a*). Log-phase cells were harvested, washed and plated in 60 mm tissue culture dishes at a density of 200 cells/dish in 5 ml of culture medium. Drugs or control vehicle were added usually as ⩽50 μl of stock solution. Resulting colonies were stained with Giemsa dye in methanol. Control dishes usually contained 75–150 colonies.

Median effect analysis of the nature of the interaction between DDF and VBL was performed as previously described (*ibid.*). This analysis yields the combination index, a measure of the extent of synergy, additivity or antagonism at various levels of cell kill (Chou & Talalay, 1984).

### *Regression analysis*

The apparent unidirectional influx and initial efflux rate in KB-GRC1 and KB-3-1 cells was linear up to 240 s ($r^2 \geq 0.90$) The rate constant for initial efflux was determined by fitting a line to the efflux data over the first 240 s of efflux. Several experiments, or runs, were performed under the same combination of cell line and condition (control, DDF or forskolin); the equation for the i-th run was $C_i(t) = A_i\ [1-k_e t]$, where $C_i(t)$ is the concentration of VBL at any time t, $A_i$ is the initial concentration of VBL at $C_{ss}$ prior to efflux, as shown by the fitted y-intercept, and $k_e$ is the rate constant for initial efflux. Simultaneous fitting of the data from all runs was done to obtain a single estimate of $k_e$; its standard error and degrees of freedom appeared in the regression output. The $r^2$ values for initial efflux fittings ranged from 0.91 to 0.96 across the various combinations of cell line and condition. Similarly, for each combination of cell line and condition, the rate constant for influx was determined by fitting a line to the influx data over the first 120 s of influx. The equation for the i-th run was $C_i(t) = A_i\ [1 + k_{in} t]$, where $C_i(t)$ is the time zero binding of VBL, as shown by the fitted y-intercept, and $k_{in}$ is the rate constant for initial uptake, i.e., influx. The $r^2$ values for influx fittings ranged from 0.94 to 0.99 across the various combinations of cell line and condition. The fitted rate constants for efflux and influx were compared between control and experimental groups using the Satterwaite modification of Student's *t* test.

### *Statistical analysis*

Unless otherwise noted, the data were expressed as the group mean ± s.d. of duplicate determinations from each of *n* experiments. The Student's *t* test for grouped data was used unless otherwise stated. In all cases, significance was at the level of $P < 0.05$.

### Results

### *Comparison of diterpenes as modulators*

Figure 1 compares the structure of forskolin, DF and DDF, which differ from each other by virtue of either one or two hydroxyl groups at the one and nine positions. Hydrophilicity increases with the number of hydroxyl groups with forskolin being most hydrophilic, DF intermediate and DDF least hydrophilic (K. Seamon, personal communication). Figure 2 shows the effect of increasing concentrations of DDF on the 1 h uptake of 6 nM [$^3$H]-VBL. We have

| COMPOUND | 1-position | 9-position |
|---|---|---|
| Forskolin | hydroxyl | hydroxyl |
| 1-Deoxyforskolin | hydrogen | hydroxyl |
| 1,9-Dideoxyforskolin | hydrogen | hydrogen |

**Figure 1** Structure of diterpene analogues.

**Figure 2** Effect of increasing concentrations of DDF on the 1 h uptake of 6 nM [$^3$H]-VBL in KB cells. Values are mean ± s.d. of 3–4 experiments. Vertical bars, s.d.; when s.d. < size of symbol, no bar is shown.

previously shown that steady-state plateau is reached by 1 h under control conditions (Shalinsky *et al.*, 1990*a*). In KB-GRC1 cells, 1–100 μM DDF produced a marked increase in the uptake with an $EC_{50}$ of about 15 μM. In contrast, DDF produced a 1.4-fold increase in the 1 h uptake in parental KB-3-1 cells. In subsequent studies, a 50 μM concentration of diterpene analogue was chosen for modulation of [$^3$H]-VBL uptake. This concentration did not alter cellular ability to exclude trypan blue. Figure 3 shows the time course of the uptake under control conditions and in the presence of DDF, DF or forskolin. Several points are evident. First, the large decrease in uptake in the KB-GRC1 relative to the KB-3-1 cell line confirmed the over-expression of a wildtype PGP in the KB-GRC1 cells. Second, the effects of the diterpenes were greater in the KB-GRC1 cells with DDF, DF and forskolin enhancing VBL content at 1 h by 13.6 ± 1.9, 8.0 ± 0.7 and 4.7 ± 2.5-fold, respectively. In contrast, the same agents produced only a 1.4 ± 0.3, 1.3 ± 0.05 and 1.2 ± 0.04-fold increase in KB-3-1 cells, respectively. These data demonstrate that DDF was the most potent of these diterpenes and that PGP was a likely target for the modulation.

The effect of dibutyryl cAMP (Robison *et al.*, 1968) on VBL uptake was examined to determine the effect of increasing cAMP content independent of any other effect that forskolin might have. Dibutyryl cAMP did not affect the 1 h uptake in either cell line at concentrations of 0.01–5.0 μM (data not shown). These concentrations have been reported to produce a maximal increase in protein kinase A activity in neoplastic cells *in vitro*, an increase that is sufficient to mediate cAMP-dependent transport of other cytotoxic drugs (Mann *et al.*, 1991). Thus, an increase in cAMP content was likely not required for modulation of VBL uptake, consistent with the fact that the magnitude of DDF's effect was substantially larger than that of forskolin.

### *Modulation of influx*

We have previously described the presence of a rapidly-acting PGP efflux transporter in KB-GRC1 cells that functions to reduce VBL uptake even during the initial seconds of exposure (Shalinsky *et al.*, 1990*b*; Shalinsky *et al.*, 1993). In a direct comparison between KB-GRC1 and KB-3-1 cells, the lowered apparent influx was associated only with the enhanced efflux in KB-GRC1, indicating that the PGP efflux activity manifested as both reduced influx and enhanced efflux. Thus, Figure 4 shows the apparent influx over the initial 120 s that is due to PGP efflux activity. The ability of PGP to reduce net accumulation was evident at times < 30 s, resulting in an approximate 2-fold decrease in the influx of VBL in KB-GRC1 cells. In KB-GRC1 cells, the diterpene pattern of modulation was the same as in Figure 3, and all three agents produced large increases in influx. However, there was a more pronounced effect of DDF and DF on [$^3$H]-VBL influx in the KB-3-1 cells than was predicated from the magnitude of the effect of these agents at 1 h. The influx data were well fit by a straight line ($r^2 > 0.94$). Table I shows that DDF, DF and forskolin produced statistically significant increases in the influx rate constant in both KB-3-1 and KB-GRC1 cells.

### *Modulation of efflux*

The results obtained with the KB-GRC1 cells were consistent with DDF and the diterpenes acting as inhibitors of PGP activity. This hypothesis was tested directly by measuring the ability of the most lipophilic compound, DDF, and the least, forskolin, to inhibit the efflux of [$^3$H]-VBL. We employed two cell models for these studies, including the KB cell lines, and mouse L cell variants, transfected with either a human wildtype, KK, or mutated and non-functional *mdr*1 gene, MM.

L cells normally express low levels of endogenous PGP (I.B. Roninson, personal communication) but none was detected by flow cytometric analysis of cells stained with the C219 antibody in the empty vector control cell line, NEO (data not shown). VBL accumulation at 1 h was the same in NEO and MM, but was reduced 3-fold in the KK cell line (data not shown). Transfection of the wildtype human *mdr*1 gene also conferred a 5–10-fold resistance to colchicine (I.B. Roninson, personal communication) and VBL in clonogenic assay in KK compared to NEO and MM cells (data not shown).

The KB cell model permitted comparison of the effect of a wildtype PGP on diterpene modulatory ability. The L cell

**Figure 3** Time course for uptake of [$^3$H]-VBL in KB cells in the presence of 50 μM diterpene analogue. Uptake was monitored in KB-GRC1 (□, control; ■, modulator) and KB-3-1 cells (○, control; ●, modulator) as indicated. Values are mean ± s.d. of 3–4 experiments (vertical bars, s.d.; no bar is shown when s.d. < size of symbol).

**Table I** Rate constants for influx of VBL over the first 120 s

| *Cell line* | *Modulator* | *Rate constant ± s.e.*[a] ( × $10^{-5}$ $sec^{-1}$) | *P-value against control* |
|---|---|---|---|
| KB-GRC1 | Control | 0.260[b] ± 0.017 | |
| | Dipyridamole[c] | 1.576 ± 0.297 | 0.002 |
| | DDF | 3.156 ± 0.143 | < 0.00001 |
| | DF | 1.683 ± 0.047 | < 0.000001 |
| | Forskolin | 0.916 ± 0.083 | < 0.0001 |
| KB-3-1 | Control | 1.634 ± 0.053 | |
| | Dipyridamole | 3.413 ± 0.372 | 0.013 |
| | DDF | 3.593 ± 0.228 | < 0.0001 |
| | DF | 4.162 ± 0.156 | < 0.00001 |
| | Forskolin | 2.318 ± 0.065 | < 0.00001 |

[a]Values are mean ± s.e. from three experiments. [b]KB-GRC1 *vs* KB-3-1 control: *P*-value = 0.000000001. [c]Dipyridamole data has been included for comparison with diterpene data. Data were obtained over 20 s (Shalinsky *et al*., 1993).

model permitted assessment of the effect of wildtype or mutated non-functional PGP on this ablity. Figure 5 shows the efflux of [$^3$H]-VBL in the KB cell lines. Examination of this figure indicates that efflux was biphasic in the KB-GRC1 cells and was more rapid and of a greater extent in KB-GRC1 than KB-3-1 cells. In KB-GRC1 cells, DDF and forskolin retarded the terminal phase of efflux. The extent of the initial inhibition was determined by regression analysis of the efflux data over the first 240 s of efflux. These data were well fit by a straight line ($r^2 \geq 0.91$). As shown in Table II, DDF significantly inhibited the initial efflux of VBL by 34% to 53% in each cell line expressing a wildtype form of PGP. Forskolin inhibited initial efflux in KB-GRC1 cells but the inhibition was not statistically significant. In KB-3-1- cells, there was no effect on initial efflux. Figure 6 shows the effect of these agents on efflux in the L cell variants. Increased efflux occurred in KK cells containing the wildtype *mdr*1 relative to MM cells containing the mutant *mdr*1, establishing the viability of the L cell model for monitoring PGP-mediated efflux. In this model, DDF inhibited initial efflux uniquely in KK cells, but DDF and forskolin retarded ter-

**Figure 4** Time course for influx of [$^3$H]-VBL in KB cells in the presence of 50 μM diterpene analogue. Uptake was monitored for 2.0 min in KB-GRC1 (□, control; ■, modulator) and KB-3-1 cells (○, control; ●, modulator) as indicated. Values are mean ± s.d. of 3–4 experiments (vertical bars, s.d.).

**Table II** Rate constants for initial efflux of VBL over the first 240 s

| *Cell line* | *Modulator* | *Rate constant* ± *s.e.*[a] *(min*$^{-1}$*)* | *P-value against control* |
|---|---|---|---|
| I. *KB Cells* | | | |
| KB-GRC1 | Control[b] | 0.122 ± 0.011 | |
| | Dipyridamole[c] | 0.74 ± 0.013 | 0.009 |
| | DDF | 0.81 ± 0.012 | 0.021 |
| | Forskolin | 0.95 ± 0.013 | 0.129 |
| KB-3-1 | Control | 0.034 ± 0.009 | |
| | Dipyridamole | 0.030 ± 0.005 | 0.193 |
| | DDF | 0.034 ± 0.012 | 0.981 |
| | Forskolin | 0.025 ± 0.009 | 0.526 |
| II. *L Cells* | | | |
| KK | Control[d] | 0.158 ± 0.023 | |
| | Dipyridamole | 0.069 ± 0.020 | 0.008 |
| | DDF | 0.074 ± 0.032 | 0.046 |
| | Forskolin | not done | |
| MM | Control | 0.073 ± 0.016 | |
| | Dipyridamole | 0.058 ± 0.008 | 0.446 |
| | DDF | 0.058 ± 0.014 | 0.487 |
| | Forskolin | 0.067 ± 0.023 | 0.846 |

[a]Values are mean ± s.e. from 3–6 experiments. [b]KB-GRC1 *vs* KB-3-1 control: *P*-value = 0.002. [c]Dipyridamole data has been included for comparison with diterpene data. Data was obtained over 2 min (Shalinsky *et al.*, 1993). [d]KK *vs* MM control: *P*-value <0.001.

**Figure 5** Efflux of [$^3$H]-VBL in KB cells in the presence of 50 μM diterpene analogue. Efflux was monitored for up to 3 h under control conditions (O), or in the presence of DDF (Δ) or forskolin (□). Values are mean ± s.d. of 3–6 experiments (vertical bars, s.d.).

**Figure 6** Efflux of [$^3$H]-VBL in mouse L cells in the presence of 50 μM diterpene analogue. Efflux was monitored for up to 90 min under control conditions (O), or in the presence of DDF (Δ), forskolin (□), or dipyridamole (■, dotted line). Values are mean ± s.d. of 3–4 experiments (vertical bars, s.d.).

minal efflux in both cell lines. Dipyridamole was used in these studies as a positive control to show the effect of an MDR modulator that acts predominantly by inhibiting PGP-mediated efflux (Shalinsky *et al.*, 1990*a*; Shalinsky *et al.*, 1991; Shalinsky *et al.*, 1993). Hence, only DDF produced a significant inhibition of initial efflux and did so in sublines expressing the wildtype *mdr1* gene, demonstrating that the mechanism of increased uptake was due to inhibition of PGP-mediated efflux. The fact that both DDF and forskolin slowed the efflux of VBL by at least 1.5-fold during the terminal phase irrespective of the presence of PGP raised the possibility that the diterpenes were altering the intracellular binding of VBL. Measurement of the extent of bound intracellular VBL demonstrated that 60% of the radiolabel was ultrafiltrable after a 1 h incubation with [$^3$H]-VBL. Forskolin and DDF did not change the level of ultrafiltrable [$^3$H]-VBL ($n = 2$, data not shown), indicating that the diterpene effect on uptake was not due to a change in a tightly-bound fraction of VBL. Overall, these data indicated that DDF was indeed inhibiting PGP efflux activity, but also pointed to other mechanisms to account for effects on terminal efflux in cells lacking functional PGP.

### *Diterpene modulation of various chemotherapeutic agents*

We further examined DDF's effect on the uptake of another substrate of PGP, [$^{14}$C]-doxorubicin, and two agents, [$^3$H]-methotrexate and [$^3$H]-DEP [a cisplatin analogue], which are not (Juranka *et al.*, 1989). These data confirmed that DDF was selective in its modulatory ability toward MDR drugs.

Figure 7 shows that similarly to its effect on [$^3$H]-VBL, DDF increased doxorubicin uptake in KB-GRC1 cells. The 35% increase indicated that the wildtype form of PGP was much more specific for VBL than doxorubicin. In contrast in KB-3-1 cells, DDF failed to alter doxorubicin uptake, nor did it elevate the uptake of [$^3$H]-methotrexate or [$^3$H]-DEP in either cell line. Thus, DDF did not affect the uptake of either of the two drugs that do not participate in the MDR phenotype.

*Assessment of non-specific diterpene effects*

Since DDF and DF stimulated VBL influx in KB-3-1 cells, further experiments were conducted to examine whether the enhancement may have been due to nonspecific membrane permeabilisation. DDF and forskolin had no affect on membrane permeability to propidium iodide as assessed by FACS analysis in the same cell suspension in which the membrane permeant, digitonin, produced a marked increase (data not shown). Liposomes were also employed as previously described (Shalinsky *et al.*, 1993) to assess the effect of forskolin on VBL uptake across a protein-free membrane. Forskolin was inactive as a modulator in liposomes over a 2 h period (data not shown). Finally, the 2 h uptake of the cation [$^3$H]-TPP$^+$ was measured in KB-3-1 cells to determine whether membrane polarisation was changed by forskolin or DDF. Control cells accumulated 2511 ± 201 pmol [$^3$H]-TPP$^+$/mg cellular protein at 2 h, but the uptake was unaffected by the diterpenes. These results indicated that the diterpenes did not nonspecifically alter membrane permeability.

**Figure 7** Ability of 50 μM DDF to enhance the 1 h uptake of radiolabelled 6 nM VBL, 1 μM doxorubicin (DOX), 10 nM methotrexate (MTX) or 5 μM DEP in KB cells. Control pmol drug accumulated mg$^{-1}$ cellular protein for each drug is shown in parentheses. Values are mean ± s.d. of 2–5 experiments.

**Figure 8** Cytotoxicity of DDF and forskolin in KB-GRC1 (○) and KB-3-1 (●) cells. The cells were exposed to increasing concentrations of drug continuously. Cytotoxicity was assessed by clonogenic assay. Values are mean ± s.d. of 4–5 experiments.

*Cytotoxic studies*

The ability of the diterpenes to selectively augment VBL uptake suggested that they would selectively enhance VBL cytotoxicity in KB-GRC1 cells. As shown in Figure 8, the sensitivity of the KB cell lines to DDF and forskolin was determined. Each cell line was more resistant to forskolin than to DDF. A non-toxic concentration of 20 μM of each modulator was used for subsequent studies. Figure 9 illustrates the ability of DDF and forskolin to enhance the cytotoxicity of VBL. In these experiments, KB-GRC1 cells were 27-fold resistant to VBL relative to KB-3-1 cells; the $IC_{50}$ values were 18.9 ± 4.5 and 0.7 ± 0.3 nM, respectively. In KB-GRC1 cells, DDF increased the sensitivity to VBL by 7-fold, whereas forskolin had a discernable 1.5-fold effect. In contrast in KB-3-1 cells, neither DDF nor forskolin affected the VBL dose response curve. Median effect analysis was used to determine the nature of the interaction between DDF and VBL in KB-GRC1 cells. Median effect analysis yielded $CI_{20}$, $CI_{50}$ and $CI_{80}$ values of 0.21 ± 0.05, 0.20 ± 0.05 and 0.18 ± 0.04, respectively ($n = 2$). CI values less than 1 indicate synergy, thus there was a highly synergistic interaction between DDF and VBL in the KB-GRC1 cells.

### Discussion

In this report, we demonstrate that the diterpene analogues, and notably DDF, selectively modulate VBL uptake, efflux and sensitivity in tumour cells over-expressing *mdr1*. These data demonstrate the feasibility of modulating sensitivity to a

**Figure 9** Clonogenic assay of vinblastine sensitivity in the presence of DDF and forskolin. KB-GRC1 cells were exposed to increasing concentrations of VBL alone (○), or to increasing concentrations of VBL in the presence of 20 μM DDF (△) or forskolin (□). KB-3-1 cells were exposed to VBL alone (●), or to increasing concentrations of VBL in the presence of 20 μM DDF (▲) or forskolin (■). Values are mean ± s.d. of three experiments (vertical bars, s.d.).

*vinca* alkaloid commonly used for the treatment of cancer. These data, when viewed in context with a previous report (Morris *et al.*, 1991) establish inhibition of PGP-mediated efflux as a primary mechanism.

Forskolin was chosen for investigation because it activates protein kinase A indirectly (Laurenza *et al.*, 1989) and alteration of protein kinase A has been shown to alter drug sensitivity (Abraham *et al.*, 1990) and phosphorylation of PGP (Meyers, 1989; Mellado & Horwitz, 1987). Forskolin also interacts with membrane-spanning proteins, such as adenylyl cyclase, and glucose and ion transporters, known to be homologous to PGP (Morris *et al.*, 1991; Krupinski *et al.*, 1989).

While PGP may be phosphorylated by protein kinase C and other kinases (Fine *et al.*, 1988; Hamada *et al.*, 1987; Staats *et al.*, 1990), the effect of PKC-activation is typically a decrease in the level of drug accumulated. We have confirmed that phorbol-treatment leads to decreased VBL uptake in KB-GRC1 cells (Shalinsky and Howell, unpublished data) suggesting that PGP-mediated efflux was stimulated by the treatment. Thus, a similar role for cAMP can be excluded because forskolin enhanced, not decreased, VBL uptake. Further studies examining PKC were not pursued since the diterpenes appear to selectively alter PKA rather than PKC pathways (Robison *et al.*, 1968; Laurenza *et al.*, 1989). However, the possibility that the diterpenes may inhibit PKC-mediated pathways to enhance VBL uptake can not be ruled out.

The approach was aimed instead at examining the involvement of cAMP based on the fact that forskolin directly activates adenylate cyclase and DDF does not (*ibid.*, *ibid.*). The data demonstrated that the diterpene augmentation of VBL uptake occurred independently of at least the protein kinase A pathway since DDF was the most potent modulator. This was supported by the lack of direct effect of dibutyryl cAMP on [$^{3}$H]-VBL uptake. These data lend support to the cAMP-independent mechanism initially postulated by Wadler & Wiernik (1988), but discount a direct role for a cAMP-dependent mechanism that may modulate PGP efflux activity at the level of the transporter itself. It therefore appears that the PKA pathway may play a prominent role in regulating the expression (Abraham *et al.*, 1990) but not the direct activity of PGP.

*Comparison of forskolin and DDF as modulators*

DDF was more potent than forskolin in enhancing uptake and was selectively active in KB-GRC1 cells. The greater increase, 13.6-fold *vs* 1.4-fold in KB-3-1 cells, suggested that DDF interacted directly with PGP to inhibit efflux activity. The selective inhibition of efflux in KB-GRC1 cells confirmed this. The same rank order for relative lipophilicity and ability to enhance VBL uptake was in good agreement with the ability of these agents to inhibit photolabelling of PGP (Morris *et al.*, 1991).

The hypothesis that VBL is a better substrate for PGP than doxorubicin in KB-GRC1 cells is substantiated by the fact that KB-GRC1 cells are 7-fold more resistant to VBL than doxorubicin (Choi *et al.*, 1988). Furthermore, while VBL may not universally be a superior substrate of PGP compared to doxorubicin, VBL has been reported to inhibit the labelling of PGP by azidopine to a greater extent than doxorubicin in some MDR cell lines (Greenberger *et al.*, 1990). Thus, our results support previously published reports.

*Modulation of influx and uptake*

An ability of PGP to efflux drug during the initial seconds of exposure has been established in KB-GRC1 (Shalinsky *et al.*, 1993) and other MDR tumour cells (Cano-Gauci *et al.*, 1990). Initial VBL influx ($\leqslant$ 120 s) in KB-GRC1 cells is energy-dependent and immediately potentiated by dipyridamole and verapamil (Shalinsky *et al.*, 1993), indicating the presence of PGP's rapid efflux activity. Furthermore, DDF has been shown to inhibit PGP-associated chloride channel activity within 30 s of exposure (Valverde *et al.*, 1992), substantiating that diterpene modulators can rapidly alter PGP function.

These diterpenes also produced increases in the rate constant for influx in KB-3-1 cells which would not be associated with PGP, but for which non-specific mechanisms have been largely excluded. It is possible that intracellular processes unrelated to membrane permeability were affected. Thus, these data implicated PGP-independent mechanisms which remain unknown.

However, the enhanced influx in KB-3-1 cells was not accompanied by large increases in the 1 h uptake, and thus it is unlikely that DDF and forskolin will be as active against drug-sensitive neoplasms. MDR modulators such as dipyridamole, verapamil and DDF produce high levels of synergy with VBL only in *mdr*1-over-expressing cells (Shalinsky *et al.*, 1990*a*; Shalinsky *et al.*, 1991). Hence the relatively large increase in the uptake in KB-GRC1 compared to KB-3-1 cells may represent the cytotoxically-relevant parameter for producing synergy.

*Modulation of efflux*

As discussed, DDF inhibited the initial efflux rate constant for VBL in both KB-GRC1 and KK cells, demonstrating selective inhibition of PGP-mediated efflux. Of note is the small effect on inhibition of initial efflux as compared to enhancement of uptake. This seems to be a general phenomenon. We have reported that dipyridamole and verapamil have very small effects on inhibiting efflux compared to their ability to enhance uptake (Shalinsky *et al.*, 1993). Similarly, phenoxazine modulators have large effects on uptake but only $\leqslant 30\%$ effects on inhibiting initial efflux (Thimmaiah *et al.*, 1990). These data suggest that some factor may hinder the ability of the efflux blocker to exert its effect when the membrane or cytoplasm is saturated with the blocker, but the basis for this observation is unclear. Thus, the small effects on efflux observed in this study were consistent with other reports. It may therefore not be surprising that forskolin failed to significantly inhibit initial influx in lieu of its smaller ability to enhance uptake *vs* DDF, and due to the fact that forskolin is a poorer antagonist of the photolabelling of PGP relative to DDF (Morris *et al.*, 1991).

DDF and forskolin inhibited terminal efflux more prominently in cells irrespective of expression of *mdr*1, yet there was no evidence for a change in the tightly-bound fraction of VBL. Every MDR modulator we have studied produces a similar pattern (Shalinsky *et al.*, 1993; unpublished data), and it would be of great interest to elucidate the mechanism(s). The mechanism(s) responsible for this effect remains to be determined.

*Selective modulation of cytotoxicity*

The ability of forskolin and DDF to enhance the uptake of VBL translated into a measurable increase in cytotoxicity selectively in the KB-GRC1 cell line. The enhanced cytotoxicity mirrored well the ability of forskolin and DDF to augment VBL uptake and to inhibit photolabelling of PGP (Morris *et al.*, 1991). Median effect analysis demonstrated a highly synergistic interaction between VBL and DDF when cytotoxic concentrations of DDF were used. Similar testing was not done in KB-3-1 cells since in the absence of an effect on $C_{ss}$, synergy was unlikely, and only additive effects have been observed between MDR modulators and VBL in previous studies in KB-3-1 cells (Shalinsky *et al.*, 1990). Forskolin may have been expected to produce less synergy with VBL than DDF, but prohibitive amounts of forskolin would have been required and thus these experiments were not done. Thus, DDF produced a large increase in sensitivity to VBL, and the synergistic nature was encouraging with regard to potential clinical utility.

*Lipophilic diterpenes as potential MDR chemosensitisers.*

DDF compared favourably with dipyridamole and verapamil in terms of its potency for enhancing the uptake of [$^3$H]-VBL. The $EC_{50}$ for DDF was 15 $\mu$M, and it was 5 and 28 $\mu$M for dipyridamole and verapamil, respectively (Shalinsky *et al.*, 1990*a*). On the other hand, the maximum stimulation of $10.8 \pm 0.3$ pmol VBL mg$^{-1}$ cellular protein by DDF at 100 $\mu$M was higher than for other agent tested to date in KB-GRC1 cells. For example, a saturating concentration of 80 $\mu$M dipyridamole and verapamil elevated the $C_{ss}$ of [$^3$H]-VBL to $6.2 \pm 0.8$ and $4.6 \pm 0.6$ pmol mg$^{-1}$, respectively (Shalinsky *et al.*, 1990*a*). Despite the larger effect of DDF on uptake, DDF produced a similar level of synergy with VBL as compared to that observed in combination with dipyridamole or verapamil (Shalinsky *et al.*, 1990*a*), but it must be noted that all three agents produced about the maximum level of synergy that can be obtained by median effect analysis ($CI_{50}$ values $\leqslant 0.2$). We can therefore conclude that each produced an excellent level of synergy in combination with VBL. Thus, DDF compared favourably at the *in vitro* level in its ability to modulate VBL toxicity. It remains to be determined whether DDF or another lipophilic diterpene will prove to be a superior chemosensitiser, and *in vivo* studies are needed to address this point. There is limited data available regarding efficacy or toxicity *in vivo* and none of the diterpenes studied herein have been approved for human use (K. Seamon, personal communication). One may reasonably expect, though, that DDF would lack the cardiotoxic side effects that limit the use of verapamil, and that lack of an effect on the cAMP pathway may also be an advantage.

### Conclusions

This report definitively links the ability of DDF to inhibit PGP-mediated efflux with enhancement of VBL sensitivity. DDF produced a selective enhancement of accumulation of [$^3$H]-VBL and sensitivity to VBL resulting in a high level of synergy with VBL in cells over-expressing *mdr*1. These effects were related to lipophilicity but were unrelated to cAMP. Thus, our data, along with previous reports (Morris *et al.*, 1991; Wadler & Wiernik, 1988), suggest that the class of lipophilic diterpene analogues may be good candidates for further study as potential MDR chemosensitisers in the anticancer therapy.

We thank Dr Igor Roninson and Mr Brian Morse for generously supplying the KB and L cell tumour lines and Centocor, Inc. for the gift of P-glycoCHEK$^R$. We also thank Dr Randolph Christen and Mr Dennis Young for performing flow cytometric studies, and Drs Shirin Khatibi and Sinil Kim for supplying the liposomes.

This work was supported by grant CA 09290 from the National Institutes of Health and grants from Boehringer-Ingelheim Inc. and Bristol-Myers Squibb Co. Dr. Shalinsky was supported in part by National Research Service Award CA 08993. This work was conducted in part by the Clayton Foundation for Research-California Division. Drs Howell and Jekunen are Clayton Foundation Investigators.

Abbreviations: $CI_{50}$ = combination index at level of 50% cell kill; $C_{ss}$ = steady state concentration; DDF = 1,9-dideoxyforskolin; DF = 1-deoxyforskolin; [$^3$H]-DEP = [$^3$H]-*cis*-dichloro(ethylenediamine)platinum (II); $EC_{50}$ = concentration of drug which produces 50% of the maximum effect; $IC_{50}$ = concentration of drug which inhibits colony formation by 50%; MDR = multidrug resistant or resistance; PGP = P-glycoprotein; PBS = phosphate buffered saline; $TPP^+$ = tetraphenylphosphonium bromide; VBL = vinblastine sulfate.

### References

ABRAHAM, I., CHIN, K.-V., GOTTESMAN, M.M., MAYO, J. & SAMPSON, K.E. (1990). Transfection of a mutant regulatory subunit gene of cAMP-dependent protein kinase causes increased drug sensitivity and decreased expression of P-glycoprotein. *Exp. Cell Res.*, **189**, 133-141.

ANDREWS, P.A., MANN, S.C., HUYNH, H.H. & ALBRIGHT, K.D. (1991). Role of the Na$^+$, K($^+$)-adenosine triphosphatase in the accumulation of cis-diaminedichloroplatinum(II) in human ovarian carcinoma cells. *Cancer Res.*, **51**, 3677-3681.

CANO-GAUCI, D.F., BUSCHE, R., TUMMLER, B. & RIORDAN, J.R. (1990). Fast kinetic analysis of drug transport in multidrug resistant cells using a pulsed quench-flow apparatus. *Biochem. Biophys. Res. Commun.*, **167**, 48–53.

CHABNER, B.A. & FOJO, A. (1989). Multidrug resistance: P-glycoprotein and its allies – the elusive foes. *J. Natl Cancer Inst.*, **81**, 910–913.

CHOI, K., CHEN, C., KRIEGLER, M. & RONINSON, I. (1988). An altered pattern of cross resistance in multi-drug resistant human cells results from spontaneous mutation in the mdr1 (P-glycoprotein) gene. *Cell*, **53**, 519–529.

CHOU, T.-C. & TALALAY, P. (1984). Quantitative analysis of dose-effect relationships: the combined effects of multiple drugs or enzyme inhibitors. *Adv. Enz. Reg.*, **22**, 27–55.

EASTMAN, A. (1983). Characterization of the adducts produced in DNA by *cis*-diamminedichloroplatinum(II) and *cis*-dichloro (ethylenediamine)platinum(II). *Biochem.*, **22**, 3927–3933.

FINE, R.L., PATEL, J. & CHABNER, B.A. (1988). Phorbol esters induce multidrug resistance in human breast cancer cells. *Proc. Natl Acad. Sci.*, **85**, 582–586.

GREENBERGER, L., HUANG YANG, C.-P., GINDIN, E. & HORWITZ, S.B. (1990). Photaffinity probes for the $\alpha_1$-adrenergic receptor and the calcium channel bind to a common domain in P-glycoprotein. *J. Biol. Chem.*, **265**, 4394–4401.

HAMADA, H., HAGIWARA, K.-I., NAKAJIMA, T. & TSURUO, T. (1987). Phosphorylation of the $M_r$ 170,000 to 180,000 glycoprotein specific to multidrug-resistant tumor cells: effects of verapamil, trifluoperazine, and phorbol esters. *Cancer Res.*, **47**, 2860–2865.

HOWELL, S.B., ISONISHI, S.I., CHRISTEN, R.D., ANDREWS, P.A. & MANN, S.C. (1991). Cellular pharmacologic strategies for overcoming drug resistance: potential applications to regional therapy. *Eur. J. Surg. Suppl.*, **561**, 45–58.

JURANKA, P.F., ZASTAWNY, R.L. & LING, V. (1989). P-glycoprotein: multidrug-resistance and a superfamily of membrane-associated transport proteins. *FASEB J.*, **3**, 2583–2592.

KRUPINSKI, J., COUSSEN, F., BAKALYAR, H.A., TANG, W.-J., FEINSTEIN, P.G., ORTH, K., SLAUGHTER, C., REED, R.R. & GILMAN, A.G. (1989). Adenylyl cyclase amino acid sequence: possible channel- or transporter-like structure. *Science*, **244**, 1558–1564.

LAURENZA, A., SUTKOWSKI, E.M. & SEAMON, K.B. (1989). Forskolin: a specific stimulator of adenylyl cyclase or a diterpene with multiple sites of action? *Tips Rev.*, **10**, 442–447.

MANN, S.C., ANDREWS, P.A. & HOWELL, S.B. (1991). Modulation of cis-diamminedichloroplatinum(II) accumulation and sensitivity by forskolin and 3-isobutyl-1-methylxanthine in sensitive and resistant human ovarian carcinoma cells. *Int. J. Cancer*, **48**, 866–872.

MELLADO, W. & HORWITZ, S.B. (1987). Phosphorylation of the multidrug resistance associated glycoprotein. *Biochem.*, **26**, 6900–6904.

MEYERS, M.B. (1989). Protein phosphorylation in multidrug resistant Chinese hamster cells. *Cancer Commun.*, **1**, 233–241.

MORRIS, D.I., SPEICHER, L.A., RUOHO, A.E., TEW, K.D. & SEAMON, K.B. (1991). Interaction of forskolin with the P-glycoprotein multidrug transporter. *Biochem.*, **30**, 8371–8379.

MORSE, B. & RONINSON, I.B. (1990). The role of nucleotide binding sites in P-glycoprotein function. *Proc. Am. Assoc. Cancer Res.*, **31**, 361 (2139).

ROBISON, R.W., BUTCHER, R.W. & SUTHERLAND, E.W. (1968). cAMP *Ann. Rev. Biochem.*, **37**, 149–175.

SHALINSKY, D.R., ANDREEFF, M. & HOWELL, S.B. (1990*a*). Modulation of drug sensitivity by dipyridamole in multidrug resistant tumor cells *in vitro*. *Cancer Res.*, **50**, 7537–7543.

SHALINSKY, D.R., CHRISTEN, R.D. & HOWELL, S.B. (1990*b*). The effect of dipyridamole and verapamil on the cellular pharmacology of vinblastine in drug resistant tumor cells. *Proc. Am. Assoc. Cancer Res.*, **31**, 360.

SHALINSKY, D.R., SLOVAK, M.L. & HOWELL, S.B. (1991). Modulation of vinblastine sensitivity by dipyridamole in multidrug resistant fibrosarcoma cells lacking *mdr*1 expression. *Br. J. Cancer*, **64**, 705–709.

SHALINSKY, D.R., JEKUNNEN, A.P., ALCARAZ, J.E., CHRISTEN, R.D., KIM, S., KHATIBI, S. & HOWELL, S.B. (1993). Regulation of initial vinblastine influx by P-glycoprotein. *Br. J. Cancer*, **67**, 30–36.

STAATA, J., MARQUARDT, D. & CENTER, M.S. (1990). Characterization of a membrane-associated protein kinase of multidrug-resistant HL60 cells which phosphorylates P-glycoprotein. *J. Biol. Chem.*, **265**, 4084–4090.

THIMMAIAH, K.N., HORTON, J.K., QIAN, X.D., BECK, W.T., HOUGHTON, J.A. & HOUGHTON, P.J. (1990). Structural determinants of phenoxazine type compounds required to modulate the accumulation of vinblastine and vincristine in multidrug-resistant cell lines. *Cancer Commun.*, **2**, 249–259.

THIMMAIAH, K.N. & SETHI, V.S. (1985). Chemical characterization of the degradation products of vinblastine dihydrogen sulfate. *Cancer Res.*, **45**, 5382–5385.

VALVERDE, M.A., HYDE, S.C. & HIGGINS, C.F. (1992). Volume regulated chloride channels associated with the human multidrug resistance P-glycoprotein. *Nature*, **355**, 830–833.

WADLER, S. & WIERNIK, P.H. (1988). Partial reversal of doxorubicin reistance by forskolin and 1,9-dideoxyforskolin in murine sarcoma S180 variants. *Cancer Res.*, **48**, 539–543.